# TRAIL, Wnt, Sonic Hedgehog, TGFβ, and miRNA Signalings Are Potential Targets for Oral Cancer Therapy

**DOI:** 10.3390/ijms18071523

**Published:** 2017-07-14

**Authors:** Ammad Ahmad Farooqi, Chih-Wen Shu, Hurng-Wern Huang, Hui-Ru Wang, Yung-Ting Chang, Sundas Fayyaz, Shyng-Shiou F. Yuan, Jen-Yang Tang, Hsueh-Wei Chang

**Affiliations:** 1 Institute of Biomedical and Genetic Engineering (IBGE), Islamabad 54000, Pakistan; ammadfarooqi@rlmclahore.com; 2 Department of Medical Education and Research, Kaohsiung Veterans General Hospital, Kaohsiung 81362, Taiwan; cwshu@vghks.gov.tw; 3 Institute of Biomedical Science, National Sun Yat-Sen University, Kaohsiung 80424, Taiwan; sting@mail.nsysu.edu.tw (H.-W.H.); whr0319@gmail.com (H.-R.W.); 4 Doctoral Degree Program in Marine Biotechnology, National Sun Yat-sen University, Kaohsiung 80424, Taiwan; poppiiyy@gmail.com; 5 Doctoral Degree Program in Marine Biotechnology, Academia Sinica, Taipei 11529, Taiwan; 6 Laboratory for Translational Oncology and Personalized Medicine, Rashid Latif Medical College, Lahore 44000, Pakistan; Sundas.khan23@yahoo.com; 7 Cancer Center, Kaohsiung Medical University Hospital, Kaohsiung Medical University, Kaohsiung 80708, Taiwan; yuanssf@ms33.hinet.net; 8 Translational Research Center, Kaohsiung Medical University Hospital, Kaohsiung Medical University, Kaohsiung 80708, Taiwan; 9 Department of Radiation Oncology, Faculty of Medicine, College of Medicine, Kaohsiung Medical University, Kaohsiung 80708, Taiwan; 10 Department of Radiation Oncology, Kaohsiung Medical University Hospital, Kaohsiung 80708, Taiwan; 11 Department of Radiation Oncology, Kaohsiung Municipal Ta-Tung Hospital, Kaohsiung 80145, Taiwan; 12 Institute of Medical Science and Technology, National Sun Yat-sen University, Kaohsiung 80424, Taiwan; 13 Department of Medical Research, Kaohsiung Medical University Hospital; Kaohsiung Medical University, Kaohsiung 80708, Taiwan; 14 Research Center for Natural Products & Drug Development, Kaohsiung Medical University, Kaohsiung 80708, Taiwan; 15 Department of Biomedical Science and Environmental Biology, Kaohsiung Medical University, Kaohsiung 80708, Taiwan

**Keywords:** TRAIL, Wnt, sonic hedgehog, TGFβ, miRNA, target therapy, oral cancer

## Abstract

Clinical studies and cancer cell models emphasize the importance of targeting therapies for oral cancer. The tumor necrosis factor-related apoptosis-inducing ligand (TRAIL) is highly expressed in cancer, and is a selective killing ligand for oral cancer. Signaling proteins in the wingless-type mouse mammary tumor virus (MMTV) integration site family (Wnt), Sonic hedgehog (SHH), and transforming growth factor β (TGFβ) pathways may regulate cell proliferation, migration, and apoptosis. Accordingly, the genes encoding these signaling proteins are potential targets for oral cancer therapy. In this review, we focus on recent advances in targeting therapies for oral cancer and discuss the gene targets within TRAIL, Wnt, SHH, and TGFβ signaling for oral cancer therapies. Oncogenic microRNAs (miRNAs) and tumor suppressor miRNAs targeting the genes encoding these signaling proteins are summarized, and the interactions between Wnt, SHH, TGFβ, and miRNAs are interpreted. With suitable combination treatments, synergistic effects are expected to improve targeting therapies for oral cancer.

## 1. Introduction

The tumor necrosis factor-related apoptosis-inducing ligand (TRAIL), also referred to as the tumor necrosis factor (ligand) superfamily, member 10 (TNFSF10), is an apoptosis-inducible cytokine [1]. TRAIL is highly expressed in cancer, and is a selective killing ligand for cancer therapy [2,3]. TRAIL may crosstalk to transforming growth factor β (TGFβ; TGFB1) in disease progression and apoptosis. For example, TRAIL can upregulate TGFβ expression to induce extracellular matrix generation in fibroblasts [4]. TRAIL-induced death-inducing signaling complex (DISC) formation for apoptosis was inhibited in TGFβ-treated human breast epithelial cells [5]. Accordingly, TRAIL and TGFβ may crosstalk to regulate migration and apoptosis. Moreover, TGFβ is also reported to crosstalk to Sonic hedgehog (SHH) to increase cyclosporine-stimulated cell proliferation of human gingival fibroblasts [6], and to increase the motility and invasiveness of gastric cancer cells [7]. TGFβ, SHH, and wingless-type MMTV integration site family (Wnt) also crosstalk to regulate mesenchymal transition (EMT) [8] in tumor progression [9]. Accordingly, these signalings are potential targets for cancer therapy. Additionally, different microRNA (miRNA) profiles may have diverse distributions and functions in regulating these signalings. The following sections aim to summarize updated literature on the roles of TRAIL, TGFβ, SHH, Wnt, and miRNAs as potential targets for oral cancer therapy.

## 2. TRAIL-Induced Intracellular Signaling in Oral Cancer

### 2.1. TRAIL May Be a Selective Killing Ligand for Cancer Cells

TRAIL binds extracellularly to death receptors (DR), and then the signal transmits intracellularly to induce extrinsic apoptosis [10]. For cancer cells to be more prone for TRAIL treatment, the DRs are commonly upregulated to induce apoptosis. Earlier, it was reported that TRAIL was expressed constitutively in normal oral epithelia, but underwent progressive loss in primary and metastatic oral squamous cell carcinomas (OSCC) [11]. Tumor cells have a higher sensitivity to TRAIL treatment for apoptosis than normal cells [10]. Accordingly, TRAIL-mediated signaling has emerged as one of the most deeply studied biological phenomena, as it allows differential apoptosis, affecting cancer cells, while leaving normal cells intact. Therefore, TRAIL may serve as a selective killing ligand targeting cancer cells [11,12].

### 2.2. TRAIL Receptor-Inducible Agents for Targeting Therapies for Oral Cancer

Mechanistically, it has been shown that the expression of TRAIL receptors can be enhanced using natural and synthetic agents (Table 1).

For example, *Smilax china* L. extract (SCE) notably enhanced the protein quantity of death receptor 5 (DR5; TNFRSF10B; tumor necrosis factor receptor superfamily, member 10b) by stabilizing it [13]. Phospho–extracellular signal-regulated kinase (pERK), a kind of mitogen-activated protein kinase (MAPK), was significantly reduced in SCE-treated oral mucoepidermoid carcinoma MC3 cells. Similarly, ERK inhibitor (PD98059), in combination with SCE, increased DR5 expression [13]. β-Phenylethyl isothiocyanate (PEITC) has previously been shown to efficiently induce apoptosis and inhibit tumor growth in mice. PEITC-treated OSCC HN22 cells induced an increase in DR5 expression via p38 MAPK. As expected, SB203580, a chemical inhibitor of p38 MAPK, drastically abrogated PEITC-induced DR5 upregulation [14]. Glycosylation inhibitor 2-deoxy-d-glucose, in combination with TRAIL, synergistically induced apoptosis in oral cancer KB cells via the upregulation of DR5 [15]. Suberoylanilide hydroxamic acid (SAHA) markedly enhanced the expressions of DR4, DR5, Fas cell surface death receptor (Fas), and the Fas ligand (FasL; FASLG) in oral cancer Ca9-22 and SAS cells [16].

Esculetin, a natural agent, efficiently induces growth arrest in oral cancer SAS cells. Moreover, DR5 has also been found to be upregulated in esculetin-treated cancer cells that consequently resulted in the sensitization of oral cancer cells to TRAIL-induced signals [17]. S-1, a fluoropyrimidine anti-oral cancer agent, in combination with TRAIL, considerably reduced tumor growth in mice xenografted with oral cancer HSC2 cells [18]. It is noteworthy that TRAIL preferentially suppressed tumor growth in B88-bearing mice, as compared to HSC2 tumor-bearing mice [18]. In addition, evidence suggests that the constitutively active Ras mutant (RasV12) expressing OSCC cells shows an increase in DR5 expression on cell surfaces. Detailed research is needed to determine the potential of TRAIL receptor-targeting therapies for constitutively active Ras mutant-expressing oral cancers [19].

## 3. Wnt Signaling and Oral Cancer

Wnt denotes *wingless* (the Drosophila segment polarity gene) and integrated or *int-1* (the vertebrate homolog) genes [20]. Two types of Wnt signaling have been proposed: a canonical Wnt pathway (β-catenin-dependent) and a non-canonical Wnt pathway (β-catenin-independent).

### 3.1. Canonical Wnt Pathway in Oral Cancer Cells

The canonical Wnt pathway (Wnt/β-catenin pathway) plays an important role in regulating cell proliferation, migration, and apoptosis [21] and is commonly deregulated in several types of cancer, such as colon [22,23], liver [24], stomach [25], breast [26], childhood T-cell acute lymphoblastic leukemia [27], and head and neck squamous cell carcinoma (HNSCC) [28]. For example, HNSCC has been reported to have abnormal expression in the canonical Wnt signaling pathway [28]. Wnt/β-catenin signaling inhibits cell detachment-mediated apoptosis (anoikis) in SCC1 cells, and promotes HNSCC-xenografting tumor growth in vivo [29]. The mRNA expressions of Wnt signaling are overexpressed in HNSCC tissues. Stem cell-enriched side population (SP) cells derived from HNSCC highly express Wnt/β-catenin signaling, but non-SP cells do not [30]. Moreover, Wnt/β-catenin signaling may regulate the epithelial to EMT expression in tumor development of laryngeal squamous cell carcinomas (SCC) [31].

The deregulation of Wnt signaling frequently occurs in OSCC and HNSCC cells. Its Wnt signaling is commonly downregulated by the methylation of several antagonists of the Wnt pathway such as dickkopf Wnt signaling pathway inhibitor 1 (*DKK1*), serine/threonine kinase 11 (*STK11*; *LKB1*), protein phosphatase 2 regulatory subunit B β (*PPP2R2B*), runt-related transcription factor 3 (*RUNX3*), secreted frizzled related protein 1 (*SFRP1*), *SFRP2*, and Wnt Inhibitory Factor 1 (*WIF-1*) genes. However, the degree of methylation of these antagonists of the Wnt pathway may express differentially for OSCC and HNSCC cells. For an example of OSCC cell lines, *WIF-1* and *SFRP2* genes are frequently methylated, but dachshund family transcription factor 1 (*DACH1*) and *DKK1* genes are methylated at a lower frequency. Similarly, the *WIF-1* gene is frequently methylated in patients with primary oropharyngeal tumors. It is noteworthy that the *WIF-1* gene methylation is correlated with shorter survival for oropharyngeal tumor patients [32]. In addition, evidence suggests that patients older than 56 years old are about five times more likely to have hypermethylation on the disheveled binding antagonist of the β catenin 2 (DACT2) promoter [33].

Surprisingly, SFRP2 mRNA is highly expressed in tumor-adjacent normal tissues, but not in tumor samples. This is further validated by cell line modeling. For example, cell proliferation is inhibited in SFRP2 overexpression of OSCC cell lines (TCA8113), and tumor growth is also reduced in mice subcutaneously inoculated with TCA8113/SFRP2 cells. Both β-catenin and glycogen synthase kinase-3 β (GSK3B; GSK-3β) are considerably enhanced in the cytoplasm and cell membrane of mice xenografted with TCA8113/SFRP2 cells [34].

The canonical Wnt pathway also regulates the migration and invasion of cancer cells. For example, galectin-3 is a prognostic marker in oral tongue carcinoma [35]. Galectin-3 overexpressing OSCC TCA8113 cells display markedly enhanced proliferation, migration and invasion [36]. Moreover, Wnt upregulation, β-catenin activation, and EMT induction are noted. Since canonical Wnt signaling may activate EMT [37], Wnt antagonists are expected to suppress EMT in cancer progression. For example, TCA8113 cells co-transfected with galectin-3 and Wnt antagonist DKK1 inhibit Wnt upregulation, β-catenin activation or EMT induction [36]. However, some Wnt antagonists sometimes display Wnt-independent functions to regulate EMT. For example, both the migration and invasion potential of DKK3-silenced OSCC cells are reduced in a Wnt-independent manner [38].

Additionally, DKK3 may play a different role in the survival of HNSCC patients compared to other types of cancer. For example, DKK3 is downregulated in various types of cancer, such as liver [39], breast [40], and prostate [41], but it is not downregulated in HNSCC cells [42]. Accordingly, downregulation of DKK3 may differ with different cancer types. Survival analysis indicates that the absence of protein expression of DKK3 results in considerably longer metastasis-free survival, disease-free survival, and overall survival of HNSCC patients [42]. Therefore, the function of Wnt antagonists in oral carcinogenesis warrants further investigation.

### 3.2. Non-Canonical Wnt Pathway in Oral Cancer Cells

The non-canonical Wnt pathway is independent of β-catenin and low-density lipoprotein-related protein 5/6 (LRP5/6) co-receptors, and contains two branches: the planar cell polarity (PCP) pathway, and the Wnt/Ca^2+^ pathway [20]. The non-canonical Wnt pathway is activated when Wnt binds to the Frizzled receptor and its LRP5/6 co-receptor. Other putative co-receptor candidates of the Frizzled receptor have been reported, including the neurotrophin receptor homolog 1 (NRH1) [43], receptor-like tyrosine kinase (Ryk) [44], protein tyrosine kinase 7 (PTK7) [45], and receptor tyrosine kinase-like orphan receptor 2 (ROR2) [46]. In OSCC cells, the non-canonical Wnt/Ca^2+^/protein kinase C (PKC) pathway is activated via the Wnt family member 5A (WNT5A), enhancing migration and invasion [47]. WNT5B expression is notably higher in SAS-LM8 cells, a highly metastatic cell line derived from OSCC SAS cells. Gene silencing of *WNT5B* considerably inhibited the formation of filopodia-like protrusive structures and migration. Stimulating SAS-LM8 cells with *WNT5B* significantly increases the formation of filopodia-like protrusions [48]. Moreover, the non-canonical Wnt/Ca^2+^ pathway can regulate Ca^2+^ release from the endoplasmic reticulum (ER) for intracellular Ca^2+^ homeostasis [20].

### 3.3. Wnt Pathway as the Target for Oral Cancer Therapy

Many small-molecule inhibitors used to specifically target Wnt signaling proteins, such as Frizzled, Disheveled, Porcupine, or Tankyrase, are well reviewed in terms of effective dosages, Chemical Abstracts Service (CAS) numbers, and targets [49]. However, their potential application in oral cancer therapy has not been investigated. Porcupine (PORCN) is a membrane-bound *O*-acyltransferase essential for palmitoylation and the secretion of Wnt ligands. LGK974, a small molecule for specific PORCN inhibition, are shown to inhibit Wnt signaling in vitro and in vivo in HNSCC HN30 cell models [50]. Honokiol, isolated from Magnolia officinalis, reduces transcription factor 4 (TCF4) and β-catenin levels in OSCC SAS cells markedly. Consequently, the target genes of β-catenin, particularly c-Myc and cyclin D1, are also reduced [51]. Accordingly, the small-molecule inhibitors and natural products for specifically targeting Wnt signaling proteins warrant further investigation in the target therapy for oral cancer in the future.

## 4. SHH Signaling and Oral Cancer

SHH, a regulator of vertebrate organogenesis [52], controls the proliferation of several types of stem cells [53,54] and cancers [55,56,57,58]. Recently, several SHH-signaling proteins were also found overexpressed in OSCC patients. For example, SHH was highly expressed in OSCC and appeared in the cytoplasm of epithelial cells in immunohistochemical analyses [59]. Both SHH and GLI family zinc finger 1 (glioma-associated oncogene homolog 1; GLI1) are notably upregulated in 74 OSCC samples of immunohistochemical analyses. GLI1 overexpression is associated with clinical staging of tumor and tumor recurrence of OSCC. The survival rate of OSCC patients with low GLI1 and SHH expression is longer than those with high expression [60]. OSCC immunohistochemistry shows the overexpression of patched 1 (PTCH1), which correlates with lymphatic metastasis [61]. Overexpression of nuclear GLI-1 is linked with tumor recurrence, lymphatic metastasis and the primary tumor size of OSCC. Moreover, PTCH1 or GLI1 overexpression is an indicative marker for poor prognosis in OSCC [61]. Circumstantial immunohistochemical evidence also suggests that higher GLI2 staining in 60/136 OSCC patients correlates with poor clinical outcomes. The survival time of GLI2-expressing patients after surgical procedures is five years [62]. By quantitative real-time PCR analysis, 30 OSCC patients were analyzed for various genes of the SHH-signaling pathway. As a result, the smoothened (Smo, a kind of frizzled class receptor), GLI1, and PTCH1 genes were highly expressed in OSCC [63].

Moreover, the role of SHH in tumor development was investigated in vivo. For example, SHH knockdown by siRNA in OSCC SAS cells failed to induce tumor angiogenesis and tumor growth when it was subcutaneously xenografted in mice [64]. Accordingly, SHH-signaling proteins were overexpressed in OSCC, and have become potential targets for oral cancer therapies.

Because SHH overexpression is a poor prognosis marker for HNSCC, as mentioned above, the development of SHH inhibitors may improve the cancer therapy of HNSCC. For example, the steroidal alkaloid cyclopamine is reported to directly bind to the smoothened in the SHH pathway to inhibit SHH signaling [65]. Cyclopamine was proved to suppress HNSCC and improve the therapeutic effect through SHH signaling [66]. Recently, several synthetic SHH antagonists were developed for clinical evaluation. GDC-0449 (vismodegib), a oral clinical trial drug, demonstrated a good efficiency and safety for basal cell carcinoma and medulloblastoma [67]. Based on preclinical models against several solid tumors (such as medulloblastoma and basal-cell carcinoma) [68,69,70], other synthetic small-molecule SMO antagonists such as SANT1, CUR-61414 [71], HhAntag-691 [72], and GDC-0449 [68] were also reported to have better performances than cyclopamine. However, the application of these SHH inhibitors in oral cancer treatment is still rare. Therefore, these putative SHH inhibitors warrant further investigation in oral cancer therapy in the future. Moreover, several natural products-derived compounds such as curcumin, epigallocatechin-3-gallate, genistein, resveratrol, zerumbone, norcantharidin, and arsenic trioxide were shown to inhibit SHH signaling [73]. Their potential application in oral cancer treatment needs reconsideration as well.

## 5. TGFβ Signaling and Oral Cancer

TGFβ is a cytokine that regulates extracellular matrix (ECM) secretion from epithelial cells [74]. TGFβ is associated with carcinogenesis-associated EMT [75,76,77]. In the TGFβ signaling pathway, the accumulation of gene mutations and overexpression of TGFβ-signaling proteins have been reported in OSCC patients. For example, out of 97 OSCC patients, mutations of the transforming growth factor β receptor 2 (*TGFBR2*) gene were noted in 21% of samples, whereas novel Smad family member 3 (*Smad3*) mutations were identified in only three cases. Accordingly, lower levels of TGFBR2 mRNA were indicative of poor overall survival and poor disease-free survival of OSCC patients [78].

By immunohistochemical staining, Smad7 was gradually increased in mild oral epithelial dysplasia moderate to severe oral epithelial dysplasia, lesions of hyperkeratosis/epithelial hyperplasia, moderately to poorly differentiated OSCC, and well-differentiated OSCC [79]. Similarly, the upregulation of Smad7 and the downregulation of Smad3, Smad 2, Smad4 and TGFβ receptor II (Tgfbr2; TβRII) in immunohistochemical staining were reported during chemically induced buccal pouch carcinogenesis [80]. By real-time quantitative RT-PCR analysis, the Smad2 negativity and Smad6 positivity were predictive of good prognoses for OSCC patients, independent of lymph nodal status, as evidenced by Cox multivariate analysis [81]. Median overall survival (mOS) was not achieved in the Smad2-negative and Smad6-positive OSCC groups of patients, but an mOS of 11.6 months was noted in a Smad2 positive/Smad6 negative subgroup [81].

Expression of E221V/N238I mutant of TGFβ receptor II (*TβRII*) gene-enhanced TGFβ has been reported to induce intracellular signaling in OSCC. More importantly, impaired lipid raft-dependent endocytosis of TβRII has been noted in mutant *TβRII*, thus highlighting the fact that considerably increased TGFβ signaling by this mutation is due to delay internalization of TβRII [82]. Inverse relationship of ADAM metallopeptidase domain 12 (ADAM12) and TGFβ3 has previously been noted by increased TGFβ3 expression in ADAM12-silenced HSC-3 cells [83].

Stimulating OSCC HSC-4 cells with TGFβ1 markedly enhanced p-SMAD2 and target genes [84]. Moreover, the zinc finger protein SNAI2 (Slug) and the integrin α3β1 were also notably increased in mRNA and protein expressions in TGFβ1-treated cells. Gene silencing of Slug by siRNA remarkably reduced TGFβ1-induced EMT and the cell migration of HSC-4 cells [84]. The role of TGFβ1 in tumor development in vivo showed that antisense TGFβ1 oligonucleotides reduced tumor growth considerably in mice xenografted with SCC9 cells [85]. Accordingly, TGFβ-signaling proteins were overexpressed in OSCC, and have become potential targets for oral cancer therapies.

## 6. miRNAs and Oral Cancer

miRNAs, primarily transcribed by RNA polymerase II, are a family of highly conserved non-protein-coding RNAs containing about 19–25 nucleotides [86]. One miRNA may modulate hundreds of targets, and multiple miRNAs may regulate one target gene [87]. Some miRNAs are oncogenic and some are tumor-suppressing in several types of cancer, such as lung, liver, breast, bladder, prostate, ovary, and kidney [88]. Different miRNA profiles may have diverse distributions and functions for different types of cancer. The function of these miRNAs acts by complementing the miRNA seed region with 3′ untranslated regions (3′ UTRs) or coding regions of target mRNAs [89], leading to mRNA degradation and subsequently decreasing their protein expressions. In this review, we compiled the targets of different miRNAs for potential oral cancer therapies in Table 2.

### 6.1. Targets of Oncogenic miRNAs for Oral Cancer Cells

Many anticancer drugs are developed by a strategy of modulating oxidative stress [90,91]. Some natural products were also reported to be anti-oral cancer cells through reactive oxygen species (ROS) modulation [92,93,94]. ROS may activate EMT transcription factors such as signal transducer and activator of transcription 3 (acute-phase response factor) (STAT3) that is activated by TGFβ, Wnt, Hedgehog, and Akt (protein kinase B, PKB) [95]. Furthermore, the p53-upregulated modulator of apoptosis (PUMA; BC2 binding component 3; BBC3) may also interact with Akt. For example, anticancer drugs such as idelalisib [96] and pazopanib [97] were reported to activate PUMA in colon cancer cells by inhibiting Akt signaling. Accordingly, Akt, STAT3, and PUMA may interact with each other. In this review, we focus on the miRNAs that regulate these signaling expressions. We briefly introduce the oncogenic miRNAs targeting Akt, STAT3, and PUMA expressions for oral cancer cells as follows:

#### 6.1.1. Akt and miR-31

Epidermal growth factor (EGF)-induced signaling is involved in the increased expression of miR-222, miR-181b and miR-31 in OSCC (HSC-3, OECM-1 and SAS) cells [98]. The chemical inhibition of a serine/threonine-specific protein kinase Akt decreases EGF-induced miR-31 expression, while cells reconstituted with Akt re-expressed miR-31 upon EGF treatment. Importantly, Akt notably enhanced the CCAAT/enhancer binding protein (C/EBP) β (C/EBPβ; CEBPB), as evidenced by the increased expression of C/EBPβ after Akt activation in oral cancer cells. Stable overexpression of the functional isoform of C/EBPβ in OSCC cells resulted in an upregulation of miR-31. Curcumin, a natural chemopreventive agent downregulated miR-31 by inhibiting Akt activation in oral cancer cells [98]. Circumstantial evidence also indicated that K14-EGFP-miR-31 transgenic mice displayed a significantly higher chemical carcinogen-mediated squamous cell tumor progression. In the squamous epithelium of transgenic mice, the expression of H2A histone family member X (H2AFX; γH2AX), a DNA double strand break marker, was notably enhanced after irradiation or treatment with chemical carcinogens. DNA repair genes, particularly Ku80 (XRCC5; X-ray repair complementing defective repair in Chinese hamster cells 5 (double-strand-break rejoining)) and PARP1 (poly (ADP-ribose) polymerase 1) were noted to be targets of miR-31 [99]. Accordingly, the Akt inhibitor may inhibit the function of oncogenic miR-31 in oral cancer therapy. Treatment with interfering oncogenic miR-31 may inhibit the oral cancer progression.

#### 6.1.2. STAT3 and miR-21

miR-21 expression is triggered by STAT3 in oral cancer TCA8113 and TSCCA cells [100]. The chemical inhibition of STAT3 using WP1066, a small molecular inhibitor that efficiently reduced miR-21 expression and re-expression of the tissue inhibitor of metalloproteinase 3 (TIMP-3), programmed cell death 4 (PDCD4) and phosphatase and tensin homolog (PTEN), was noted. WP1066 also induced tumor regression in OSCC-xenografted mice as well [100]. Accordingly, STAT3 inhibitor may inhibit the function of oncogenic miR-21 in oral cancer therapy. Treatment with interfering oncogenic miR-21 may inhibit the oral tumor growth.

#### 6.1.3. PUMA and miR-222

PUMA is a pro-apoptotic protein that is negatively regulated by miR-222 in oral cancer TCA8113 and UM1 cells [101]. Targeting miR-222 considerably improves PUMA expression and produces a higher apoptotic rate in cells [101]. Accordingly, PUMA activator may inhibit the function of oncogenic miR-222 in oral cancer therapy. Treatment with interfering oncogenic miR-222 may induce apoptosis to inhibit the oral carcinogenesis.

### 6.2. Targets of Tumor Suppressor miRNAs for Oral Cancer Cells

**Figure 1:**
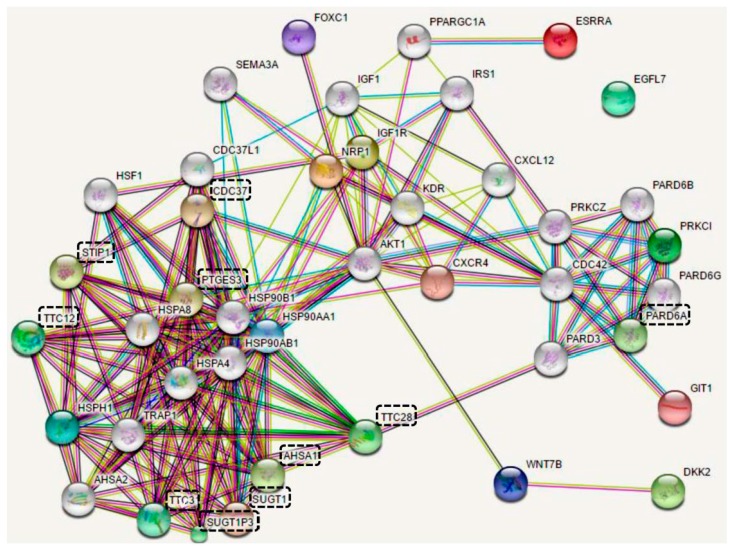
The predicted protein–protein interaction for the tumor suppressor oncogenic microRNA (miRNA) targets in Table 2. This network was constructed using STRING version 10.5 [102]. All the protein symbols carry the official name of HUPO. Proteins encoded by tumor suppressor miRNAs targets in Table 2 do not have any boxes, i.e., ERRRA, NRP1, IGF1R, DKK2, PRKCI, EGFL7, HSPH1, HSP90AA1, WNT7B, FOXC1, GIT1, and CXCR4. Ten proteins within the dash-lined box (SUGT1, CDC37, PTGES3, STIP1, AHSA1, PARD6A, TTC28, SUGT1P3, TTC12, and TTC31) indicate the predicted functional partners connecting to the proteins encoded by tumor suppressor miRNAs targets. Only EFGL7 is unable to find the possible interacting proteins under the current version of STRING. The meanings associated with each of the colored lines are the same as mentioned in STRING, including (1) known interaction from curated databases or experimentally determined, (2) predicted interactions from gene neighborhood, gene fusion, or gene co-occurrence, and (3) others (text mining, co-expression, or protein homology). In brief, proteins with lines connecting to others indicate the existence of possible interactions.

Several targets of tumor suppressor miRNAs have been summarized in Table 2. These targets were retrieved from the literature. Due to the complex interactions, it remains difficult to understand the relationship and crosstalk between their encoding proteins. The possible protein–protein interaction for these tumor suppressor miRNAs targets were predicted (Figure 1) using the bioinformatic tool, STRING, version 10.5 [102]. The interactions between these targets contain several closely related pathways and form a network. Although 10 functional partners were predicted (dash-lined box), we only focus on the targets of tumor suppressor miRNAs (no box). In oral cancer cells, several tumor suppressor miRNAs and their corresponding targets are summarized as follows (Section 6.2.1, Section 6.2.2, Section 6.2.3, Section 6.2.4, Section 6.2.5, Section 6.2.6, Section 6.2.7, Section 6.2.8, Section 6.2.9, Section 6.2.10 and Section 6.2.11).

#### 6.2.1. Neuropilin-1 (NP-1; NRP1) and miR-338

miR-338 is frequently downregulated in oral cancer TCA8113 and SCC15 cells, and the enforced expression of miR-338 resulted in the inhibition of colony formation, migration, proliferation and invasion [103]. Neuropilin-1 (NP-1) is a target of miR-338, and enforced expression of NP-1 impaired those miR-338-exerted inhibitory effects on oral cancer cells [103]. Accordingly, NP-1 inhibitors may activate the function of tumor suppressor miR-222 in oral cancer therapy. Treatment with increasing tumor suppressor miR-338 expression may inhibit the metastasis of oral cancer cells.

#### 6.2.2. Forkhead Box C1 (FOXC1) and miR-639

Ectopic expression of miR-639 in tongue cancer CAL 27 and SCC9 cells considerably inhibited TGFβ (TGFB1; transforming growth factor, β 1)-induced EMT [104]. However, targeting inhibition of miR-639 in tongue cancer cells promoted TGFβ-induced EMT. FOXC1 was reported as a target of miR-639, as evidenced by EMT in FOXC1 overexpressing cancer cells and the loss of TGFβ-induced EMT in FOXC1-silenced cells [104]. Accordingly, miR-639 may inhibit FOXC1 expression. FOXC1 inhibitor may suppress the EMT processes in oral cancer therapy. Treatment with increasing tumor suppressor miR-639 expression may inhibit the metastasis of oral cancer cells.

#### 6.2.3. Protein Kinase CI (PRKCI) and miR-219

PRKCI, a known target of miR-219, is noted to enhance colony formation, migration, cell proliferation and the invasion of tongue squamous cell carcinoma (TSCC) cells. Overexpression of miR-219 in tongue cancer CAL 27 and SCC15 cells markedly inhibited carcinogenesis [105]. Accordingly, miR-219 may inhibit PRKCI expression. PRKCI inhibitor may suppress the migration and invasion processes in oral cancer therapy. Treatment with increasing tumor suppressor miR-219 expression may inhibit metastasis of oral carcinogenesis.

#### 6.2.4. WNT7B, miR-329, and miR-410

It has recently been shown that the stable ectopic expression of WNT7B in miR-329 or miR-410 overexpressing OSCC cells restored its invasive and proliferation potential [106]. The combination of the HDAC inhibitor and the demethylation agent considerably enhanced miR-410 and miR-329 in oral cancer cells. However, betel nut alkaloids, particularly arecoline, dramatically reduced miR-410 and miR-329 [106]. Accordingly, miR-329 or miR-410 may inhibit WNT7B expression. WNT7B inhibitor may suppress the migration and invasion processes in oral cancer therapy. Treatment with increasing tumor suppressor miR-329 or miR-410 expression may inhibit metastasis of oral carcinogenesis.

#### 6.2.5. Heat Shock Proteins (HSP) and miR-27a

Certain miRNAs were shown to enhance hyperthermia-induced cell death [114]. Oral cancer cells transfected with miR-27a mimic displayed notably improved thermal sensitivity [107]. Moreover, it was shown that overexpression of heat shock proteins induced resistance against hyperthermia. Both Hsp90 (HSP90AA1; heat shock protein 90 kDa α (cytosolic), class A member 1) and Hsp110 (HSPH1; heat shock 105 kDa/110 kDa protein 1) were markedly reduced in miR-27a mimic-transfected oral cancer HSC-4 cells [107]. Accordingly, miR-27a may inhibit Hsp90 and Hsp110 expressions. Hsp90 and Hsp110 inhibitors may suppress the resistance against hyperthermia therapy. Treatment with increasing tumor suppressor miR-27a expression may improve the thermal sensitivity of oral cancer therapy.

#### 6.2.6. Estrogen-Related Receptor α (ERRα; ESRRA) and miR-125a

The transcription factor ERRα is involved in the regulation of different target genes in cancer [115,116]. ERRα is a target of miR-125a and ERRα levels were drastically reduced in miR-125a-overexpressing oral cancer SCC084 and SCC131 cells [108]. Moreover, miR-125a-expressing oral cancer cells induced significant regression of tumors in xenografted mice. Accordingly, miR-125a may inhibit ERRα expression. ERRα inhibitors may suppress tumor growth. Treatment with elevated tumor suppressor miR-125a may reduce tumor growth in oral cancer therapy.

#### 6.2.7. Epidermal Growth Factor-Like Domain 7 (EGFL7) and miR-126

miR-126 was identified within intron 7 of *EGFL7* genes [117]. miR-126 overexpression inhibited EGFL7 in OSCC-15 cells remarkably. The secretion of two key regulators of angiogenesis, basic fibroblast growth factor (bFGF) and vascular endothelial growth factor (VEGF), were also inhibited [109]. Accordingly, miR-126 may inhibit EGFL7 expression. EGFL7 inhibitor may suppress angiogenesis. Treatment with elevated tumor suppressor miR-126 expression may reduce angiogenesis in oral cancer therapy.

#### 6.2.8. Insulin-Like Growth Factor I Receptor (IGF1R) and miR-99a

IGF1R is a target of miR-99a, and is frequently overexpressed in oral cancer and the IGF induced repression of miR-99a [88]. miR-99a overexpression inhibited IGF1R expression and suppressed migration and invasion in vitro in oral cancer OEC-M1 and CGHNC9 cells. Mechanistically, it has been shown that treatment of oral cancer cells with inhibitors of MAPK and PI3K impaired IGF1-induced repression of miR-99a [110]. Accordingly, miR-99a mutually controls IGF1R expression, and miR-99a may inhibit IGF1R expression. IGF1R inhibitor may suppress the migration and invasion of oral cancer cells. Treatment with increasing tumor suppressor miR-99a expression may reduce metastasis in oral cancer therapy.

#### 6.2.9. G-Protein-Coupled Receptor Kinase-Interacting Protein 1 (GIT1) and miR-491-5p

GIT1, frequently overexpressed in oral cancer, is a target of miR-491-5p [111]. miR-491-5p overexpression resulted in declining GIT1 levels and a marked decrease in the migration and lung metastasis of OSCC cells [111]. Detailed mechanistic insights revealed that miR-491-5p overexpressed GIT1-silenced OSCC cells. This indicated a reduction in focal adhesions, and a decline in the steady-state levels of phospho-paxillin, paxillin, and phospho-focal adhesion kinase (pFAK) [111]. Moreover, EGF/EGFR-induced intracellular signaling through downstream effectors, including ERK1/2 and matrix metalloproteinases (MMP) 2/9, was also repressed in OSCC cells. Accordingly, miR-491-5p may inhibit GIT1 expression. GIT1 inhibitor may suppress the migration and focal adhesions. Treatment with increasing tumor suppressor miR-491-5p expression may reduce metastasis in oral cancer therapy.

#### 6.2.10. C-X-C Motif Chemokine Receptor 4 (CXCR4) and miR-9

Proliferation potential was notably reduced in miR-9-overexpressing oral cancer TCA8113 and SCC9 cells with highly aggressive manners [112]. *CXCR4* gene is a target of miR-9. CXCR4 is quantitatively controlled by miR-9 and the activation of CXCR4 with its ligand, notably increasing Wnt-induced intracellular signaling [112]. Curcumin, isolated from the rhizome of Curcuma longa, induced upregulation of miR-9 in SCC9 cells. Expression levels of β-catenin, GSK-3β and phospho-GSK-3β were notably enhanced in curcumin-treated SCC9 cells [118]. Accordingly, miR-9 may inhibit *CXCR4* expression. *CXCR4* inhibitor may inhibit oral cancer cell proliferation. Treatment with increasing tumor suppressor miR-9 expression may display antiproliferation in oral cancer therapy.

#### 6.2.11. DKK2 and miR-21

Dickkopf Wnt-signaling pathway inhibitor 2 (DKK2) is negatively regulated by miR-21. miR-21 silenced cells did not show significant migration in OSCC SCC25 cells [113]. miR-21 negatively regulated DKK2 and negatively promoted invasions via the Wnt/β-catenin pathway. Accordingly, miR-21 may inhibit DKK2 expression. DKK2 inhibitor may suppress the migration processes in oral cancer therapy. Treatment with increasing tumor suppressor miR-21 expression may inhibit the metastasis of oral cancer cells.

## 7. Interactions between TRAIL, Wnt, SHH, TGFβ, and miRNA Signaling Proteins in Cancer Cells

### 7.1. TRAIL-Induced Apoptosis and ER Stress

Cellular oxidative stress may cause the accumulation of protein misfolding and lead to endoplasmic reticulum (ER) stress. Several drugs and natural products have been reported to modulate the ER stress and oxidative stress in cancer therapy [119]. Detailed investigations into the relationship between ER stress and TRAIL-induced apoptosis signaling in many cancer cell lines have been reported [120,121,122,123,124]. ER stress may also be connected to apoptosis [125]. TRAIL and its receptor have been detected in several oral cancer cells (HSC-2, HSC-3, HSC-4, Ca9-22, and KB) [126]. After exogenous TRAIL treatment for 16 hours, KB cells were found to be the most sensitive, while the others were relatively more resistant. Accordingly, most oral cancer cells are resistant to TRAIL-induced cytotoxicity, suggesting that extra treatment in combination with TRAIL may be needed to overcome its TRAIL resistance.

In addition, the modulation of ER stress has been reported to regulate apoptosis. For example, suppression of eIF2α dephosphorylation by the salubrinal (ER stress inhibitor; a specific eIF2α phosphorylation-inducing agent) improves the TRAIL-induced apoptosis in human hepatoma HepG2 cells [127]. The ER stress inducer thapsigargin sensitizes human esophageal cancer EC109 and TE12 cells to TRAIL-induced apoptosis via AMPK activation [128]. Accordingly, the role of ER stress in TRAIL-induced apoptosis remains controversial and may depend on tissue-specific or TRAIL resistant characters. Therefore, modulation of ER stress signaling may affect the clinical efficacy of TRAIL-targeting cancer therapies. Several natural products have also been reported to induce ER stress in oral cancer cells [129,130,131,132,133]. Further investigation is warranted into the role TRAIL plays in these ER stress-modulating drugs and the future use of natural products for TRAIL-targeting oral cancer therapies.

### 7.2. miRNA and TRAIL Signaling

Several TRAIL receptor-targeting agents have been used to develop drugs in clinical trials [11]. Adenovirus-mediated TRAIL expression was developed to improve TRAIL delivery for cancer therapy. However, adenovirus delivery systems may also affect normal cells because of a lack of tumor specificity [134]. Overcoming this nonspecific targeting has emerged as an important issue for adenovirus delivery of TRAIL. A differential expression profile of miRNAs between bladder cancer and normal cells has been reported [135]. For example, expression of miR-1, miR-133 and miR-218 was downregulated in bladder cancer, but not in normal bladder mucosal tissue [134]. The recombinant adenovirus system containing TRAIL and miRNA response elements (MREs) of miR-1, miR-133 and miR-218 was constructed and was shown to be bladder cancer specific in regulating TRAIL expression in vitro and in vivo [134]. Similarly, tumor-targeting TRAIL expression by miRNA to inhibit tumor growth or induce apoptosis was also reported in melanoma [136], prostate [137], breast [138], lung [139], and ovarian [140] cancer cells by different miRNAs.

However, some miRNAs are sensitive to TRAIL-induced apoptosis of certain types of cancer cells, but some miRNAs are resistant to them [87,141,142,143]. For example, miR-29 (liver cancer), miR-130a (liver cancer), miR-212 (liver cancer), and miR-185 (breast and kidney cancers) are sensitive to TRAIL-induced apoptosis in these cancer cells, whereas miR-25 (liver cancer), miR-222 (liver and lung cancers), and miR-221 (lung, liver, and bladder cancers) are resistant to TRAIL-induced apoptosis [142].

Mechanistically, some miRNAs targeting the TRAIL signaling proteins were reported to modulate TRAIL resistance in cancer cells. miRNAs have been reported to regulate apoptosis in antiapoptic or proapoptotic effects through DR signaling [144]. When looking at the example of the antiapoptic effect, miR-25 targets DR4 and the hedgehog signaling may upregulate miR-25 expression for apoptosis resistance to TRAIL-induced cholangiocarcinoma [145]. Here, miR135a-3p may upregulate DR5 expression to sensitize TRAIL-induced apoptosis in tanshinone I-treated prostate cancer cells [146]. Therefore, the identification of miRNAs involving the modulation of resistance to TRAIL-induced apoptosis in oral cancer cells warrants further investigation.

### 7.3. Other Complex Crosstalk

TGFβ may rapidly induce expression of Kruppel-like transcription factors GLI2 and GLI1 [147], which function as Hedgehog effector molecules [148]. Accordingly, there is crosstalk between TGFβ and hedgehog signaling in cancer. Moreover, a link between EMT and GLI-mediated regulation was reported [149]. Similarly, crosstalk between TGFβ, SHH, and Wnt [8] regulates EMT in carcinogenesis and diseases in general [9]. GLI2, GLI1, TGFβ, SHH, and Wnt show rather complex crosstalk.

In addition, miRNAs also target and regulate *TGFβ*, *SHH*, and *Wnt* gene expressions [150]. For example, several miRNAs targeting TGFβ signaling in breast cancer metastasis [151] and in the inflammatory microenvironment of cancer are well reviewed [152]. Several miRNAs with oncogenic or tumor suppressive functions are known to interact with Wnt-signalling pathways in carcinogenesis [153,154,155] and disease [156]. miRNAs also interact with hedgehog signaling in disease [157]. However, these interactions remain unclear in oral carcinogenesis and therapy. Accordingly, the interactions between TRAIL, Wnt, SHH, TGFβ, and miRNA signaling proteins in oral cancer therapy warrant further investigation.

## 8. Conclusions

Data obtained from cancer cell lines and clinical studies have considerably improved our understanding of the potential for targeting therapies in oral cancer treatment. TRAIL is a selective killing ligand for cancer, and several TRAIL receptor agents have been developed for use in OSCC. Wnt, SHH, and TGFβ pathway signaling proteins are also suitable targets for oral cancer therapy. Oncogenic miRNAs and tumor suppressor miRNAs with specific targets display diverse functions in oral cancer therapy through regulating carcinogenesis, migration, and invasion in vitro and in vivo. Moreover, the interactions between TRAIL, Wnt, SHH, TGFβ, and miRNAs are complex in regulating carcinogenesis. Synergistic effects from the combination of treatments may improve the targeting therapies for oral cancer.

## Figures and Tables

**Table 1:** Drugs and natural products that regulate the tumor necrosis factor-related apoptosis-inducing ligand (TRAIL) signaling pathway.

Drugs/Natural Products	Gene Expression	OSCC Cell Lines	References
*Smilax china* L. extract (SCE)	1. enhances *DR5* 2. reduces *pERK*	MC3	[13]
β-Phenylethyl isothiocyanate (PEITC)	1. enhances *DR5*	HN22	[14]
2-deoxy-d-glucose	1. enhances *DR5* when combined with TRAIL	KB	[15]
Suberoylanilide hydroxamic acid (SAHA)	1. enhances *DR4*, *DR5*, *Fas*, and the *Fas* ligand	Ca9-22, SAS	[16]
Esculetin	1. enhances *DR5*	SAS	[17]
S-1 (fluoropyrimidine anti-oral cancer agent)	1. reduces tumor growth of OSCC-xenografting mice when combined with TRAIL	HSC2-xenografting mice	[18]

**Table 2:** miRNAs and their target genes in oral cancer cells.

miRNAs	Target Genes	OSCC Cell Lines	References
Oncogenic miRNAs			
miR-31	*Akt*	HSC-3, OECM-1, SAS	[98]
miR-21	*STAT3*	TCA8113, TSCCA	[100]
miR-222	*PUMA*	TCA8113, UM1	[101]
Tumor suppressor miRNAs			
miR-338	*NP-1*	TCA8113, SCC15	[103]
miR-639	*FOXC1*	CAL 27, SCC9	[104]
miR-219	*PRKCI*	CAL 27, SCC15	[105]
miR-410	*WNT7B*	DOK, FaDu, OC-3, OEC-M1, SCC4, SCC9, SCC15, SCC25, Tw2.6, YD-15	[106]
miR-27a	*Hsp90*, *Hsp110*	HSC-4	[107]
miR-125a	*ERRα*	SCC084, SCC131	[108]
miR-126	*EGFL7*	OSCC-15	[109]
miR-99a	*IGF1R*	CGHNC9, OC-3, OEC-M1, TW2.6, FaDu, KB, SCC4, SCC15, SCC9, SCC25, UT-MUC-1, YD-15, DOK, Tu183, UMSCC1, HSC3	[110]
miR-491-5p	*GIT1*	CGHNC9, SAS, SCC25, OECM-1, OC-3	[111]
miR-9	*CXCR4*	TCA8113, SCC9	[112]
miR-21	*DKK2*	SCC25	[113]

## Abbreviations

TRAIL	Tumor necrosis factor-related apoptosis-inducing ligand
Wnt	*Wingless* type MMTV integration site family
SHH	Sonic hedgehog
TGFβ	Transforming growth factor β
DR	Death receptors
OSCC	Oral squamous cell carcinomas
ERK	Extracellular signal regulated kinase
MAPK	Mitogen-activated protein kinase
HNSCC	Head and neck squamous cell carcinomas
EMT	Epithelial to mesenchymal transition
ECM	Extracellular matrix
ER stress	Endoplasmic reticulum stress
